# Diagnostic and Prognostic Potential of CXCL9 and CXCL10 Chemokines in Alcohol-Associated Liver Disease

**DOI:** 10.3390/ijms262311717

**Published:** 2025-12-03

**Authors:** Agnieszka Szczerbinska, Jacek Rolinski, Agata Surdacka, Halina Cichoz-Lach

**Affiliations:** 1 Doctoral School, Medical University of Lublin, 20-950 Lublin, Poland; 2 Department of Clinical Immunology, Medical University of Lublin, 20-950 Lublin, Poland; 3 Department of Gastroenterology and Hepatology with Endoscopy Unit, Medical University of Lublin, 20-090 Lublin, Poland

**Keywords:** alcohol-associated liver disease, C-X-C motif chemokines, CXCL9/MIG, CXCL10/IP-10, CXCL16

## Abstract

Alcohol-associated liver disease (ALD) is the leading cause of liver-related mortality. In ALD, excessive inflammatory response may induce a massive loss of hepatocytes and lead to irreversible liver damage with progressive fibrosis. Chemokines stimulate the migration of immune cells to the site of inflammation and contribute to the inflammatory cascade that may result in organ failure. We aimed to investigate blood concentrations of CXCL9/MIG, CXCL10/IP-10, and CXCL16 chemokines and their diagnostic and prognostic significance in patients with ALD. In a prospective observational study, 88 individuals were recruited, including 63 patients with ALD (44 men and 19 women, aged 48.49 ± 10.88) and 25 healthy control volunteers matched for age, sex, and ethnicity. In blood samples, concentrations of CXCL9/MIG, CXCL10/IP-10, and CXCL16 were measured using immunoenzymatic ELISAs. Correlations were examined between CXCL levels and (a) traditional inflammatory markers (C-reactive protein, white blood cell count, neutrophil count, lymphocyte count, and neutrophil-to-lymphocyte ratio-NLR) and (b) liver dysfunction severity scores: Child–Turcotte–Pugh (CTP), MELD-NA, MELD 3.0, and modified Maddrey’s discriminant function (mDF). Patients’ survival within 30 days of hospital admission was recorded for analysis. CXCL capabilities in predicting the severity of liver dysfunction and ALD outcome were validated. ALD patients showed significant systemic upregulation of all studied chemokines compared to the control group. Patients with advanced liver disease, classified as MELD-Na ≥ 20, MELD3.0 > 19, and CTP class C, as well as poor short-term outcomes, presented with significantly higher CXCL9 and CXCL10 levels compared to their counterparts. ALD non-survivors had significantly higher concentrations of all studied CXCLs in comparison to controls. Positive correlations between CXCL16 and CRP, leukocytosis, neutrophils, and NLR were confirmed (0.67; 0.46; 0.48; 0.54, respectively). Although none of the chemokines correlated with ALT activity, CXCL9, CXCL10, and CXCL16 showed positive correlations with bilirubin and alkaline phosphatase and inverse correlations with albumin levels. Our findings revealed the diagnostic and prognostic value of the studied CXCLs in ALD. In particular, CXCL9 and CXCL10 may have potential for discrimination of severe liver dysfunction and poor short-term prognosis. Further multicenter studies are required to confirm our results.

## 1. Introduction

Chemotactic cytokines, also called chemokines, are small proteins (~8–14 kDa) that play a key role in the activation and migration of immune cells—mainly leucocytes—to different sites of injury and inflammation. So far, over fifty human chemokines have been identified and classified into four families based on their structure, specifically the number and location of the first two cysteine residues at the end of the amino chain [1,2]. Human chemokines are secreted either constitutively, where they are responsible for maintaining homeostasis, or in response to infection or inflammation [2]. These critical determinants of tissue biology can bind to their corresponding G protein-coupled receptors expressed on target immune and non-immune cells and induce a certain response. There are conventional chemokine receptors (cCKRs) engaged in cell migration, adhesion, and other biological responses, and atypical chemokine receptors (ACKRs) vital for chemokine localization, distribution, and abundance [1]. This allows for controlling the activation of only those cells that express a specific receptor and can restore the particular organ or tissue balance. Some chemokines have a direct impact on angiogenesis and the tissue microenvironment [3] and may guide cellular interactions during both anti-tumor and pro-tumorigenic responses [4]. The composition of secreted chemokines in a tissue is influenced by various trigger factors, the character of immune response, and the genetic susceptibility of an affected patient. Interestingly, naturally induced autoantibodies targeting predominant chemokines at autoimmune and tumor sites were described. They are produced against the highly expressed chemokines in the area as a consequence of B-cell tolerance loss. Although the underlying mechanism of their synthesis is not fully understood, it may be beneficial for the host [5].

Since chemokine-driven cell migration may contribute to the development of disorders of various origins (i.e., autoimmunity, allergy, chronic inflammatory disease, atherosclerosis, cancer) triggered by immunological and/or inflammatory response, its disruption has therapeutic potential. Recent research has highlighted the importance of several C-X-C motif chemokines in mediating hepatic inflammation, immune cell recruitment, and fibrogenesis in ALD. Experimental studies in knockout mice have clearly shown that a single chemokine or chemokine receptor may transform the phenotype of liver disease in vivo [6,7,8]. Their effects can vary from unfavorable (i.e., pro-inflammatory, profibrogenic) to favorable (i.e., antifibrotic) [9,10]. The issue requires complete clarification before targeted treatment becomes available. In the liver, chemokines not only regulate hepatocyte function but can also control the behavior of nonparenchymal resident cells, including hepatic stellate cells, liver macrophages (i.e., Kupffer cells), endothelial cells, and circulating immune cells [11,12].

The outcomes of distinct chemokine action and their receptors differ in liver illnesses. In recent years, much effort has been made to clarify chemokines’ contribution to the inception and preservation of liver damage. However, their role in ALD requires further investigation. In the majority of ALD patients, ethanol triggers an enhanced immune and inflammatory response, leading to elevated circulating levels of pro-inflammatory cytokines and chemokines. As a result, activation and recruitment of peripheral immune cells to the liver occur. Also, the interplay between cytokines/chemokines, damage-associated molecular patterns (DAMPs), and the gut–liver axis amplifies liver injury and disease progression [13,14,15,16]. The cytokine and chemokine storm and hepatic inflammatory infiltration lead to oxidative stress with a release of reactive oxygen species (ROS) and other inflammatory mediators that contribute to progressive tissue injury and liver function deterioration, perpetuating a vicious circle [15].

ALD remains a major global health concern, with a spectrum ranging from steatosis to cirrhosis and hepatocellular carcinoma, leading to alarmingly high rates of disability and premature mortality [17]. Since the disease pathogenesis remains obscure, there is still no approved therapy and abstinence remains the treatment of choice in all stages of ALD. Liver transplantation is the only therapeutic option that improves prognosis in ALD non-responders to medical therapy, and it should be considered in well-selected patients with severe alcohol-associated hepatitis (AH) and decompensated ALD [18].

ALD is often asymptomatic until advanced stages, and ~75% of patients present only after decompensated cirrhosis has developed [19]. Early diagnosis and prognostic stratification could enable timely interventions (e.g., abstinence programs and targeted therapies) to halt disease progression [20]. Unfortunately, traditional laboratory parameters lack sensitivity/specificity in early ALD. Therefore, there is a pressing demand for cost-effective and reliable strategies to detect symptomless stages of alcohol-induced hepatic damage [21]. In light of growing evidence that chemokines are key players in the pathogenesis of ALD, further investigation is warranted. Understanding the mechanisms by which CXCLs contribute to ALD is crucial for developing future targeted therapies and improving diagnostic and prognostic strategies [22,23,24].

Accordingly, our study aimed to explore blood concentrations of selected C-X-C motif chemokines in patients with ALD and analysis of their potential associations with the severity of liver function impairment and traditional mediators of inflammation. We investigated monokine induced by gamma interferon (MIG)/C-X-C motif ligand 9 (CXCL9), interferon gamma-induced protein 10 (IP-10)/C-X-C motif ligand 10 (CXCL10), and C-X-C motif ligand 16 (CXCL16).

Studies in both patients and animal models [25,26,27,28] justify selecting CXCL9, CXCL10, and CXCL16 for investigation in ALD, as their levels and activities correlate with disease severity, immune cell infiltration, and fibrotic outcome. CXCL9 is involved in ALD pathogenesis by orchestrating T-cell recruitment and IFN-gamma-mediated responses [29]; this can limit fibrosis under some conditions [25], yet high CXCL9 also reflects severe disease (portal hypertension, organ dysfunction) and portends poor prognosis [30]. CXCL10 was one of several CXC chemokines upregulated in liver tissue, correlating with the degree of neutrophil infiltration and portal hypertension [27]. A transcriptomic analysis identified CXCL10 as a distinct, upregulated chemokine in livers of patients with AH but not in other liver diseases [31]. In a mouse model of alcohol-induced liver injury, a Chinese herbal formula that inhibited CXCL16 expression lowered liver enzymes, steatosis, and fibrogenesis, while exogenous CXCL16 administration reversed the improvements, restoring liver injury and fibrosis in the model [26]. By analyzing these chemokines in ALD, one can gain insight into the immune pathogenesis of alcohol-induced liver damage and potentially identify prognostic biomarkers or novel therapeutic targets to improve ALD outcomes.

The additional exploratory endpoint included an assessment of the potential predictive value of the studied chemokines for the 30-day disease outcome. Our prospective observational cohort study was conducted in the Gastroenterology and Hepatology Department with the Endoscopic Unit in cooperation with the Department of Clinical Immunology, Medical University of Lublin, Poland.

## 2. Results

### 2.1. The Study Cohort Characteristics

Our prospective study included 88 individuals, including 63 patients with ALD, predominantly males (44 men, 19 women, aged 48.49 ± 10.88), and 25 healthy controls matched for age, sex, and ethnicity. Given the single-center study in Lublin, Poland, all subjects were Caucasian. The comparison of demographic data of the study and control groups is displayed in Table 1.

Since women are more prone to the negative effects of alcohol [32,33], we began the study with the evaluation of the characteristics of the study group based on patient gender. However, no significant clinical or biochemical differences were found between females and males in our cohort. The results are presented in Table 2.

Stratification of ALD patients based on their gender, disease severity by CTP, MELD-Na, MELD 3.0, and mDF scores, hepatic-related complications at admission (ascites, hepatic encephalopathy, esophageal varices), and survival within 30 days of follow-up was performed. In total, 16 of 63 (25.4%) patients with ALD were diagnosed with probable alcohol-associated hepatitis (AH) based on the clinical National Institute on Alcohol Abuse and Alcoholism (NIAAA) criteria (i.e., heavy alcohol consumption, typical liver tests, no confounding factors, no liver biopsy-proven) [34]. In total, 7 of 63 (11.11%) patients with ALD (1 of 19 women, 6 of 44 men; *p* = 0.6643) died within 30 days of follow-up due to liver-related complications. The majority of non-survivors presented with clinically diagnosed AH (AH: 6 of 16 versus ALD: 1 of 46, *p* = 0.0031). The aforementioned data are presented in Table 2 and Table 3.

### 2.2. Comparison of CXCL Chemokine Concentrations in Patients with ALD and the Control Group

Significant systemic upregulation of all studied molecules was observed in patients with ALD compared to the control group. Of note, all ALD survivors and non-survivors had significantly higher blood levels of all studied CXCLs in comparison to controls. The results of these comparisons are presented in Table 4.

### 2.3. Correlation Analyses of the Studied CXCL Chemokines

**Figure 1:**
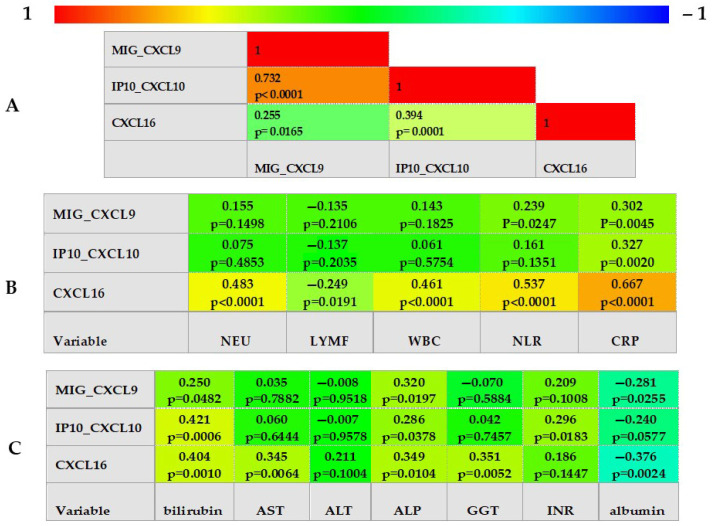
(**A**) The correlogram of CXCLs in ALD patients. (**B**) The correlogram of CXCLs and conventional inflammatory markers in ALD patients. CRP-C—reactive protein; LYMF—lymphocytes; NEU—neutrophils; NLR—neutrophil-to-lymphocyte ratio; WBC—white blood cells. (**C**) The correlogram of CXCLs and liver function parameters in ALD patients. ALP—alkaline phosphatase; ALT—alanine aminotransferase; AST—aspartate aminotransferase; GGT—gamma-glutamyl transpeptidase; INR—international normalized ratio. (**A**–**C**) Spearman’s rank correlation coefficient.

The next step of our study included correlation calculations. All studied CXCLs revealed positive correlations with each other; however, the strongest one was confirmed for CXCL9 and CXCL10. Subsequently, we checked correlations of the three studied molecules with traditional inflammatory markers, such as C-reactive protein (CRP), white blood cells (WBC), neutrophils (NEU), lymphocytes (LYMF), neutrophil-to-lymphocyte ratio (NLR), and liver function parameters. Among the studied chemokines, CXCL16 revealed the strongest association with all traditional markers of inflammation, particularly high with CRP, which was also positively correlated with the other two CXCLs. Although none of the chemokines correlated with ALT activity, all three CXCLs revealed positive correlations with bilirubin and alkaline phosphatase (ALP), but inverse correlations with albumin levels. CXCL16 correlated with most liver parameters besides ALT and INR. The results are presented in Figure 1.

### 2.4. Comparison of Blood CXCL Concentrations Among ALD Patients with Various Severities of Liver Dysfunction

ALD patients with severe liver dysfunction, classified as CTP class C, MELD-Na ≥ 20, and MELD 3.0 > 19, presented with significantly higher levels of CXCL9 and CXCL10, but CXCL16 concentrations did not differ significantly in these subgroups.

Based on our statistical analysis, we were indeed able to identify distinct concentration ranges for CXCL9 and CXCL10 that correspond to varying liver disease severity as defined by CTP, MELD-Na, and MELD 3.0.

#### 2.4.1. Stratification by Child–Turcotte–Pugh (CTP) Class

We observed a stepwise increase in CXCL9 and CXCL10 median concentrations from Class A to Class C, with statistically significant differences driven primarily by the comparison between compensated (Class A) and decompensated (Class C) patients as follows:

➢ CXCL9 (MIG):
  ✓ Class A (Compensated): Median 70.40 pg/mL (IQR: 47.92–104.81).
  ✓ Class C (Decompensated): Median 114.80 pg/mL (IQR: 77.49–207.71).
  ✓ Significance: *p* = 0.0244, the difference was significant, with post hoc analysis (Dunn’s test) confirming a significant elevation in Class C compared to Class A.
➢ CXCL10 (IP-10):
  ✓ Class A (Compensated): Median 126.49 pg/mL (IQR: 62.06–202.42).
  ✓ Class C (Decompensated): Median 333.52 pg/mL (IQR: 164.35–480.73).
  ✓ Significance: *p* = 0.0066, CXCL10 showed the most robust difference), with levels in Class C nearly triple those of Class A.
➢ CXCL16:
  ✓ Class A (Compensated): Median 2.41 pg/mL (IQR: 2.00–2.87).
  ✓ Class C (Decompensated): Median 2.99 pg/mL (IQR: 2.23–3.99).
  ✓ Significance: *p* = 0.0962, while there was a slight numerical trend upward (Class A vs. Class C), the difference was not statistically significant, indicating that CXCL16 levels did not differ in patients with various grades of liver dysfunction.

#### 2.4.2. Stratification by MELD-Na and MELD 3.0 Scores

We utilized clinically relevant cut-offs (MELD-Na ≥ 20 and the updated MELD 3.0 > 19), which are validated proxies for the severity of liver dysfunction and mortality risk. Both CXCL9 and CXCL10 significantly differed between these subgroups.

##### Stratification by MELD-Na Score (Severity Cut-Off: 20)

➢ CXCL9 (MIG):
  ✓ Lower severity (MELD-Na < 20): Median 73.10 pg/mL (IQR: 54.43–102.68).
  ✓ Advanced severity: (MELD-Na ≥ 20): Median 114.80 pg/mL (IQR: 72.78–223.70).
  ✓ Significance: *p* = 0.0101.
➢ CXCL10 (IP-10):
  ✓ Lower severity (MELD-Na < 20): Median 180.96 pg/mL (IQR: 113.26–268.54).
  ✓ Advanced severity: (MELD-Na ≥ 20): Median 333.52 pg/mL (IQR: 143.04–486.13).
  ✓ Significance: *p* = 0.0089.
➢ CXCL16:
  ✓ Lower severity (MELD-Na < 20): Median 2.65 pg/mL (IQR: 2.18–3.27).
  ✓ Advanced severity: Median 3.12 pg/mL (IQR: 2.49–4.19).
  ✓ Significance: *p* = 0.0590.

Consistent with the CTP results, CXCL16 levels between high and low MELD score subgroups did not reach statistical significance.

##### Stratification by MELD 3.0 Score (Severity Cut-Off: 19)

➢ CXCL9 (MIG):
  ✓ Lower severity: (MELD 3.0 ≤ 19): 71.19 pg/mL (IQR: 54.27–100.56).
  ✓ Advanced severity: (MELD 3.0 > 19): Median 114.80 pg/mL (IQR: 77.49–235.28).
  ✓ Significance: *p* = 0.0029.
➢ CXCL10 (IP-10):
  ✓ Lower severity: (MELD 3.0 ≤ 19): Median 173.35 pg/mL (IQR: 112.81–265.55).
  ✓ Advanced severity: (MELD 3.0 > 19): Median 306.50 pg/mL (IQR: 164.35–480.73).
  ✓ Significance: *p* = 0.0046.
➢ CXCL16:
  ✓ Lower severity: (MELD 3.0 ≤ 19): Median 2.72 ng/mL (IQR: 2.20–3.30).
  ✓ Advanced severity: (MELD 3.0 > 19): Median 3.11 ng/mL (IQR: 2.31–4.00)
  ✓ Significance: *p* = 0.1773

Consistent with the CTP and MELD-Na results, CXCL16 levels between high and low MELD 3.0 subgroups did not reach statistical significance, suggesting it may be a less sensitive marker for staging severity than CXCL9 or CXCL10.

**Figure 2:**
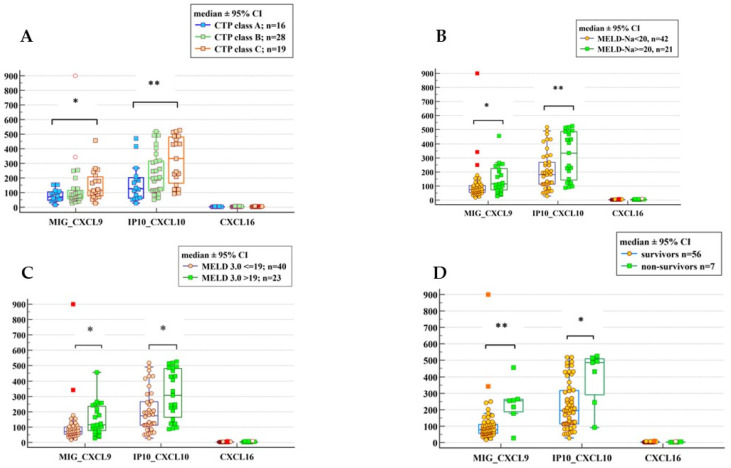
Comparison of the studied CXCL concentrations (pg/mL) in ALD patients with (**A**) different CTP classes; (**B**) MELD-Na ≥ 20 versus <20; (**C**) MELD 3.0 > 19 versus ≤19; (**D**) survivors versus non-survivors. *Y*-axis label: Chemokine concentration (pg/mL); *X*-axis label: Chemokine type. A–Kruskal–Wallis test; B, C, D—Mann–Whitney test; * *p* < 0.05; ** *p* < 0.01.

Our results indicate that CXCL10 appears to be the most sensitive marker for severity, with median levels rising from 126.49 pg/mL in early-stage disease (CTP A) to 333.52 pg/mL in advanced decompensation (CTP C, MELD-Na ≥ 20). CXCL9 follows a similarly significant pattern. However, CXCL16 did not demonstrate significant discriminatory power between disease severity stages in ALD. The results are shown in Figure 2.

Next, we assessed the discriminating capabilities of the studied CXCLs for the severity of liver dysfunction and mDF. The predictive values of the studied CXCLs and the standard clinical scores (MELD 3.0 and MELD-Na) for the short-term survival were also checked.

The performances of CXCL9 and CXCL10 in the discrimination for MELD-NA ≥ 20 and MELD 3.0 > 19 were fair but did not differ significantly. CXCL16’s ability to discriminate severe disease was poor. The performances of all studied CXCLs in the discrimination for mDF ≥ 32, which is an indicator of severe AH, were not satisfactory either.

**Figure 3:**
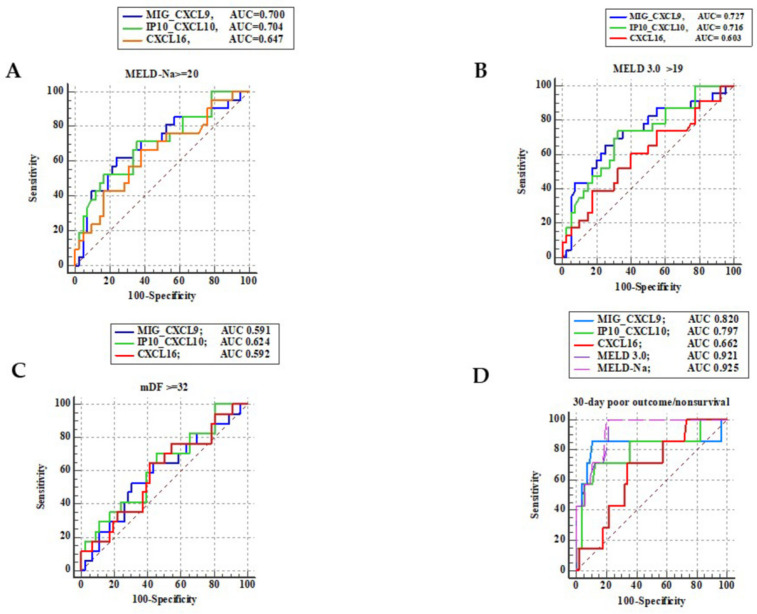
Accuracy of the studied CXCLs for discrimination of: (**A**) MELD-Na ≥ 20; (**B**) MELD 3.0 > 19; (**C**) mDF ≥ 32; (**D**) 30-day poor outcome in patients with ALD.

The results are shown in Figure 3.

### 2.5. Comparison of CXCL Chemokine Concentrations in ALD Patients Based on Their Outcomes Within 30 Days of Follow-Up

The concentrations of CXCL9 and CXCL10, in contrast to CXCL16, were significantly higher in the blood of ALD non-survivors in comparison to ALD survivors (Figure 2D). The ability of CXCLs to discriminate poor short-term survival in comparison to the standard clinical scores (MELD 3.0 and MELD-Na) was also checked. Comparison of AUCs revealed that both MELD 3.0 and MELD-Na performed better in the discrimination of 30-day outcomes; however, except for CXCL16 (*p* = 0.0054 and *p* = 0.0061, respectively), the differences with the other two CXCLs were not significant. The results of the aforementioned analyses are illustrated in Figure 3D.

Next, we applied logistic regression for the identification of independent predictors of poor ALD outcomes. Two models were created to test the performance of the studied CXCLs in comparison to MELD 3.0 and MELD-Na. Only the standard clinical scores (i.e., MELD 3.0 and MELD-Na) turned out to be independent predictors of patient non-survival, while none of the CXCLs reached statistical significance. Although all studied CXCLs are elevated in non-survivors, they do not add independent predictive value over MELD scores. The results are presented in Table 5.

## 3. Discussion

Accumulating evidence indicates that CXCL chemokines are critical regulators of immune and inflammatory responses and central mediators in the pathogenesis of chronic liver disease. While chemokine dysregulation has been reported in patients with metabolic dysfunction-associated steatotic liver disease, MASLD (formerly known as nonalcoholic fatty liver disease- NAFLD), viral and autoimmune hepatitis data in ALD remain scarce. Despite robust preclinical data, clinical translation remains limited. Most studies are observational or based on animal models, and there is a need for well-designed clinical trials to validate chemokine-targeted therapies and biomarker utility [35,36]. The heterogeneity of ALD and overlapping features with other liver diseases also pose challenges for biomarker specificity and therapeutic targeting [37]. Nevertheless, upregulation of CXCLs was observed in human and animal models of alcohol-associated liver injury, mediating neutrophil and macrophage recruitment, hepatic stellate cell activation, and fibrogenesis [14,38,39]. In line with those reports, our prospective observational study demonstrated that systemic concentrations of MIG_CXCL9, IP-10_CXCL10, and CXCL16 were significantly elevated in patients with ALD compared to matched healthy controls. Among these chemokines, CXCL9 and CXCL10 showed the strongest association with advanced severity of liver dysfunction as defined by CTP, MELD-Na, and MELD 3.0 scores and were predictive of 30-day mortality. Previous experimental studies have already suggested that blockade of CXCL10 with a neutralizing antibody reduces hepatic inflammation and fibrosis, underscoring its pathogenic potential [40,41]. CXCL9 and CXCL10, which are both interferon-gamma-inducible chemokines, seem to be particularly relevant in this context as they promote Th1-mediated immune responses and enhance inflammatory cell trafficking to the site of injury. Danger-associated molecular patterns (DAMPs), released from damaged hepatocytes, activate pattern recognition receptors, further stimulating chemokine production and perpetuating the inflammatory cycle [22,23,37,42]. Simultaneously, the gut–liver axis, through increased intestinal permeability and translocation of bacterial products, further contributes to chemokine-driven liver injury [43,44,45]. Emerging evidence suggests that circulating levels of CXCL9, CXCL10, and CXCL16, as well as their presence in extracellular vesicles, might serve as non-invasive biomarkers for ALD diagnosis, disease staging, and prognosis [15,46,47,48,49,50,51]. Targeting these chemokines or their signaling pathways has shown promise in preclinical models, with potential to attenuate liver inflammation and fibrosis [22,26,36,37].

Our findings support CXCLs’ role in driving progressive hepatocellular damage and indicate that they may serve as clinically relevant biomarkers and potential therapeutic targets in ALD. Moreover, our study extends the insights into the clinical setting, showing, for the first time, that CXCL9 and CXCL10 not only reflect ALD severity but also may predict short-term prognosis in patients with ALD. These findings underscore that CXCL9 and CXCL10 are critical inflammatory triggers, helping recruit immune cells that mediate liver injury and setting the stage for subsequent fibrogenesis and conversion to cirrhosis. Of note, Berres et al. [30] reported that patients with liver cirrhosis revealed increased CXCL9 concentrations; those with lower levels in portal and hepatic veins survive longer after transjugular intrahepatic portosystemic shunts (TIPS). Portal CXCL9 concentrations dropped after TIPS placement, showing that interventions leading to hepatic decompression can modulate chemokine levels. These observations reflect CXCL9’s prognostic value. Better survival after TIPS insertion was also associated with decreased CXCL10 levels [52]. Inhibition of CXCL10 attenuated macrophage M1 polarization and reduced liver fibrosis [53]. Therefore, both molecules could serve as biomarkers to guide therapeutic decisions in ALD patients.

Since CXCL10 has been shown to enhance the activity of effector CD4^+^ and CD8^+^ T cells and facilitate their migration to sites of inflammation (including tumor microenvironments), inhibiting this chemokine may offer therapeutic benefits for several T cell–driven autoimmune disorders, such as psoriasis, rheumatoid arthritis (RA) [43,44], inflammatory bowel disease [54], and type I diabetes (T1DM) [55], but also ALD as revealed in our study. Notably, we found that all studied CXCLs positively correlated with bilirubin and alkaline phosphatase, which were previously reported as poor prognostic indicators in ALD [56,57]. A lack of CXCLs correlation with aminotransferase activities observed in our study may indicate their closer link to systemic inflammation rather than hepatocyte necrosis in ALD. The development of systemic pro-inflammatory cascade and immune cell recruitment is a common feature of excessive alcohol consumption and leads to the immune imbalance with increased susceptibility to infections. Ethanol-induced cytokine/chemokine release and production of reactive oxygen species (ROS) cross far beyond hepatic damage, affecting other organs and tissues in the body. Our findings confirm systemic immune involvement and are consistent with the current consensus that ALD is a systemic illness characterized by numerous extrahepatic manifestations [58,59].

Notably, there is evidence that CXCL9 may also exert antifibrotic effects by suppressing the expression of α-SMA, Collagen-III, and Collagen-I in the liver, which highlights the need for further comprehensive research before chemokine-based therapy can be introduced in clinical settings [25,60].

The third studied chemokine, CXCL16, also seems to be engaged in ALD pathogenesis. It revealed significant correlations with traditional markers of inflammation, particularly with CRP, which confirms its pro-inflammatory potential. In contrast to the other two CXCLs, its blood concentrations did not differ in patients with various grades of liver dysfunction, nor in ALD survivors and non-survivors. However, CXCL16 levels were higher in both ALD survivors and non-survivors in comparison to controls.

As already mentioned, CXCL9 and CXCL10 are IFN-gamma-inducible CXCR3 ligands critical for recruitment of Th1-type CD8^+ T cells, NK cells, and other pro-inflammatory effectors into the liver [12,29]. By contrast, CXCL16 signals via CXCR6 to attract hepatic NKT cells and certain memory T cells. Soon after liver injury, macrophages in the liver upregulate CXCL16, which facilitates the swift recruitment of NKT cells to areas of damage. CXCL16/CXCR6- driven accumulation of NKT cells acts as a key early amplifier of inflammatory signaling during the initial phase of the immune response [1,7,12]. In summary, CXCL9 and CXCL10 function as wide-scope indicators of systemic inflammatory activation, while, in contrast, CXCL16 is tied to the CXCR6-NKT pathway, which plays a key role in early immune surveillance and the regulation of fibrogenesis. This distinction helps explain our findings: CXCL9 and CXCL10, reflecting a diffuse Th1-skewed injury, rise steeply with worsening ALD and predict mortality, whereas CXCL16 (and its CXCR6-NKT pathway) may not vary as tightly with acute severity. In fact, prior work in cirrhosis showed that higher CXCL9 levels predict poorer survival [30], supporting our findings that CXCL9 and CXCL10 better capture the injurious immune milieu in advanced ALD.

Several reports indicated that the CXCL16–CXCR6 axis orchestrated the recruitment of lymphocytes expressing CXCR6 (e.g., NKT, NK, and CD8^+^ T cells) to inflamed hepatic tissue. Upregulation of CXCL16 was found in viral hepatitis, MASLD, and ALD, where it promoted fibrosis development [49,50,61]. In contrast to our findings, CXCL16 blood levels have not been widely reported in ALD patients. Nevertheless, its robust upregulation in hepatic tissue has already been demonstrated [61]. Clear evidence that the CXCL16/CXCR6 axis drives liver fibrogenesis comes from Wehr et al. [7], who observed that CXCR6^−^/^−^ mice were strongly protected from hepatic inflammation and fibrosis, showing markedly reduced NKT cell and macrophage numbers and less collagen deposition compared with wild-type controls. In ALD, data on soluble CXCL16 are scarce. The study by Liu et al. [38] implies that patients with AH present with a systemic increase in CXCL16 synthesis, which is in agreement with our findings. Results obtained in our study indicate that CXCL16 could join the panel of inflammatory cytokines (e.g., IL-8, IL-6) as an activity biomarker. Given the acute-on-chronic nature of AH, a chemokine like CXCL16 might reflect the burst of immune cell trafficking into the liver. This remains an area for further investigation.

Last, but not least, there is growing evidence that CXCL9/CXCL10/CXCR3 and CXCL16/CXCR6 are involved in tumor pathogenesis, so they probably represent pleiotropic chemokines at the crossroads of immunity and carcinogenesis [28,62,63].

Moreover, there is a clear rationale for comparing CXCL9, CXCL10, and CXCL16 with established inflammatory cytokines such as IL-6, IL-8, and TNF-α, which are well known for their value in predicting ALD severity. Based on the current literature and our findings, CXCLs are likely complementary rather than strictly “better”, as they capture distinct pathophysiological aspects of the disease. While TNF alfa, IL-6, and IL-8 (CXCL8) are markers of the innate immune “storm” (neutrophil-related), CXCL9 and CXCL10 reflect the adaptive immune response (Th1/lymphocytic) as well as hemodynamic severity (portal hypertension) [29,64,65,66]. The former ones are representative of acute inflammation (e.g., alcohol-associated hepatitis), so they can be non-specifically elevated in systemic inflammatory response (infections, sepsis), which is common in ALD chronic phase. The latter ones might perform “better” in specific contexts. In our study, CXCL9 and CXCL10 were associated with mortality and correlated with MELD scores, likely because they reflect the underlying severity of ALD and portal hypertension, whereas IL-6 and IL-8 mainly capture the superimposed acute inflammatory trigger. On the other hand, CXCL16 is unique as a scavenger receptor on macrophages and endothelial cells. Its expression is closely linked to the uptake of oxidized lipids and bacterial products (gut–liver axis) and the activation of hepatic stellate cells [29,49,61]. Therefore, it potentially offers superior insight into the fibrogenic power and the metabolic/gut-derived mechanisms of liver injury [67,68]. Summing up, we do not consider these chemokines as replacements for IL-6, IL-8, and TNF alfa, but as complementary components in a possible multi-marker diagnostic panel. Combining these markers likely provides more accurate risk stratification for patients with ALD, covering both the acute precipitant and the chronic underlying severity.

Taken together, the studied chemokines may act as important amplifiers of liver injury by facilitating the infiltration of pro-inflammatory cells and perpetuating the inflammatory milieu. The interplay between chemokines, DAMPs, and the gut–liver axis underscores the complexity of ALD pathogenesis and highlights multiple potential therapeutic targets. CXCLs’ roles as biomarkers and therapeutic targets are promising but require further clinical confirmation. Accurate risk stratification in ALD is a persistent challenge. Traditional prognostic scores (CTP, MELD-Na, MELD 3.0, mDF) rely mainly on routine biochemical and clinical parameters, which may not capture the dynamic immune-inflammatory process driving disease progression. Therefore, unlike CTP and MELD scores, which reflects liver synthetic and excretory function, CXCL9 and CXCL10 indicate inflammatory burden. The predictive accuracy of CXCL9 and CXCL10 for short-term survival suggests that chemokine profiling could complement conventional prognostic tools and improve early identification of high-risk patients. CXCL9/CXCL10 have potential as adjunct prognostic biomarkers. Emerging data in liver failure syndromes support the above statements. As an example, a recent study on HBV-related acute-on-chronic liver failure (ACLF) found that another CXC chemokine, which was CXCL1, predicted 28-day mortality almost as well as MELD (AUC 0.74 vs. 0.75) [69]. Therefore, incorporating CXCL9 and CXCL10 into prognostic models could improve risk stratification. A combined CTP or MELD-plus-chemokine score might more accurately identify high-risk ALD patients than traditional systems alone, so CXCLs, as noninvasive and linked to immune activity assays, could complement existing scores. This has particular relevance for guiding decisions regarding patient monitoring, liver transplantation referral, and emerging immunomodulatory therapies.

Several limitations of our study should be acknowledged. First, this was a single-center study with a relatively small sample size; particularly, the number of non-survivors was limited. Second, the follow-up period was confined to 30 days, and longer-term prognostic implications remain unknown. Third, the potential confounding impact of self-reported alcohol consumption could not be fully controlled [70]. Nevertheless, self-reported abstinence was found to be reliable in a recent clinical trial [71].

Future multicenter studies with larger patient cohorts are required to validate our findings and to establish recommended cut-off values of studied CXCLs for clinical application. Longitudinal assessments of chemokine dynamics in relation to abstinence, relapse, and therapeutic interventions will further define their clinical utility. Moreover, experimental studies targeting CXCL9/CXCL10/CXCL16 pathways may help establish their real potential as therapeutic targets in ALD with efficacy and safety assessment. Despite advances in research, gaps remain in understanding the precise roles of individual CXCLs and their interactions with other inflammatory mediators. There is also a scarcity of clinical trial evidence on CXCL-targeted therapies in ALD. This subject requires additional research to explain all aspects of CXCLs signaling to make them useful as disease biomarkers and a potential therapeutic target for ALD. Given the growing interest in immunotherapy for severe ALD, our study sets the stage for larger studies to confirm whether blocking or monitoring C-X-C chemokine pathways can improve ALD outcomes.

## 4. Materials and Methods

### 4.1. Participants’ Recruitment

Consecutive patients aged ≥18 years, diagnosed with ALD, and admitted to our department in a 14-month timeframe were eligible for participation in the study. All the patients signed a written informed consent, and their medical history was reviewed. Alcohol consumption over the preceding 3–6 months was assessed based on data derived from an interview conducted with patients themselves and members of their families [72,73]. Moreover, the AUDIT-C (alcohol use disorders identification test—consumption) questionnaire [74] was applied to verify alcohol exposure. The rules of enrollment included evaluation of inclusion and exclusion criteria, which are presented below.

Inclusion criteria consisted of the following:

1. Patient agreement and signature of the informed consent.
2. Age group: Adults aged 18 years and above.
3. Confirmation of active alcohol consumption exceeding 40 g/day for men and 20 g/day for women during the last 6–12 months before enrollment, as well as positive results of the AUDIT-C evaluation, i.e., a score of 4 or more in men and 3 or more in women.
4. Physical examination revealing the presence of findings suggestive of chronic liver disease (hepatomegaly, a firm liver edge, splenomegaly, sarcopenia, palmar erythema, parotid enlargement, jaundice, etc.).
5. Laboratory liver function tests consistent with alcohol-associated liver injury, including moderately elevated ALT and AST levels with a reversed de Ritis ratio (AST/ALT) above 2, significantly increased gamma-glutamyl transpeptidase (GGT).
6. Elimination of other etiologies of liver disease (viral infection, immune-related disorders, drug-induced liver disease, Wilson’s disease, hemochromatosis, etc.).

Exclusion criteria consisted of the following:

1. Lack of written approval/agreement to participate in the study.
2. Individuals younger than 18 years.
3. Steroid/immunosuppressant treatment in the last 6 months before study enrollment.
4. Use of non-steroidal anti-inflammatory drugs on a long-term basis and/or documented hepatotoxic effects of other medications within the 6 months prior to study enrollment.
5. Blood and/or blood products transfusion in the last 6 months before study enrollment.
6. Serious comorbidities that preclude an assessment and are associated with poor prognosis and/or require complex clinical management that might influence the study outcome (e.g., tumors, respiratory/circulatory failure, chronic renal failure, complicated and unstable diabetes mellitus or other endocrinopathies, advanced hematologic illness, etc.).
7. Pregnancy.

### 4.2. Methods of the Study Group Assessment

Following the study protocol, all scheduled procedures were performed and data required for further analyses were recorded within 48 h of hospital admission. Patient data were anonymized. At least 24 h of alcohol abstinence was required before blood samples were obtained for laboratory investigations.

After clinical evaluation on admission, patients with ALD were divided into subgroups according to their gender, degree of liver failure, and disease complications, including liver decompensation. Portal hypertension manifestations in the upper gastrointestinal tract (i.e., esophageal varices) were revealed by gastroscopy. The presence of fluid accumulation in the peritoneal cavity was confirmed by abdominal USG examination. West Heaven criteria were applied for hepatic encephalopathy (HE) assessment [75]. Cholestasis was identified based on alkaline phosphatase (ALP) levels exceeding 1.5 times the upper limit of normal (ULN) and gamma-glutamyltranspeptidase (GGT) levels greater than three times the ULN, in accordance with the guidelines of the European Association for the Study of the Liver (EASL) [76]. Every patient with symptoms of cholestasis had their anti-mitochondrial antibodies (AMA) checked to exclude primary biliary cholangitis (PBC). Other possible causes of bile duct obstruction (e.g., gallstone disease) were ruled out by USG scanning. Kidney dysfunction was defined by elevated blood creatinine levels (above the upper limit of the normal range, that is, 1.3 mg/dL). Any further health concerns were explained based on imaging evaluation, including computed tomography (CT) and/or magnetic resonance (MR) scanning, and/or duplex Doppler ultrasound (USG) scanning. ALD patients were followed for 30 days in an outpatient clinic or during any subsequent hospitalization if required.

Volunteers with normal liver function tests, normal abdominal USG scans, and no health problems were recruited to the control group. They reported either abstinence or alcohol consumption not exceeding 20 g/day.

### 4.3. Laboratory Tests and Examinations of Blood CXCL Concentrations

All standard laboratory investigations, including hepatic function tests, were performed in the Central Laboratory of the University Hospital No.4 in Lublin.

For each patient enrolled in the study, the following parameters were checked:

➢ Liver function blood tests, including liver enzymes, i.e., alanine aminotransferase (ALT), aspartate aminotransferase (ASP), alkaline phosphatase (ALP), gamma-glutamyltransferase (GGT), total bilirubin (T-bilirubin), albumin, prothrombin time (PT), and the international normalized ratio (INR).
➢ Complete blood count (CBC).
➢ Kidney function tests (creatinine and urea), electrolyte levels (sodium and potassium).
➢ Conventional markers of inflammation, including C-reactive protein (CRP) level, leucocytes (white blood cells count, WBC), neutrophils (NEU) count, and neutrophil to lymphocyte ratio (NLR).
➢ Additional markers to exclude other than ALD etiologies of chronic liver disease, including HBs antigen, anti-HBc antibodies, anti-HCV antibodies, and HCV RNA if required, autoantibodies, tests for Wilson’s disease, and hemochromatosis.

Based on the obtained lab results, the severity of liver dysfunction was determined. The scores of Child–Turcotte–Pugh (CTP), model of end-stage liver disease (MELD), and Maddrey discriminant function (mDF) were computed using online calculators [77,78,79,80].

Blood CXCL concentrations were measured using commercial enzyme-linked immunosorbent assay (ELISA) kits (Thermo Fisher Scientific-Invitrogen, USA) strictly following the manufacturer’s protocols provided with each kit. The following kits were used:

- Human MIG/CXCL9 ELISA Kit (cat. #EHCXCL9; sensitivity 20 pg/mL, assay range 20–6000 pg/mL);
- Human IP-10 (CXCL10) ELISA Kit (cat. # KAC2361; sensitivity < 2 pg/mL, assay range 7.8–500 pg/mL);
- Human CXCL16 ELISA Kit (cat. # EHCXCL16; sensitivity 3 pg/mL, assay range 3–2000 pg/mL).

According to the study protocol, five milliliters of peripheral blood were gathered from the ulnar vein (EDTA tubes; Medlab, London, UK) of patients and individuals from the control group after an overnight fast. Within 4 h of collection, blood samples were centrifuged for 10 min at 3000× *g* to separate the clot and then, without delay, were aliquoted and stored at (−80) °C for further analyses. Each specimen underwent a single freeze–thaw cycle to minimize potential analyte degradation and variability. Immunological examinations were carried out under the supervision of Prof. Jacek Roliński at the Department of Clinical Immunology of the Medical University of Lublin, utilizing the specialized equipment available in that facility, which ensured compliance with the department’s standard operating procedures and quality control guidelines.

### 4.4. Statistical Analysis

MedCalc^®^ Statistical Software version 23.3.7 (MedCalc Software Ltd., Ostend, Belgium; https://www.medcalc.org; 25 August 2025) was applied for statistical analyses. The distribution of data was checked using the Kolmogorov–Smirnov test. Due to the data set’s skewed values, the Mann–Whitney U test was applied for comparisons of quantitative variables, and they are presented as medians with the interquartile range. Categorical variables are displayed as totals and proportions, and for their comparisons, either the Chi-square test or Fisher’s exact test was used as appropriate. Differences in chemokine concentrations in patients with three CTP classes were analyzed using Kruskal–Wallis and post hoc Dunn tests. The relationships between the routine blood parameters and the studied chemokines were examined using Spearman’s correlation test. Receiver Operating Characteristics (ROCs) and Area Under the Curves (AUCs) were used for estimation and comparison of predictive CXCL values for the discrimination of advanced liver dysfunction and poor patient short-term prognosis. Logistic regression was applied for the selection of independent predictors of poor 30-day outcome in the ALD group. The level of significance was set at a two-tailed *p* < 0.05. In the Figures, significance levels are marked using one asterisk for *p* = 0.05, two for *p* = 0.01, and three for *p* < 0.001. Cases with random missing data were excluded from the analysis.

## 5. Conclusions

The results of our study indicate potential roles of CXCL9/CXCL10/CXCL16 chemokines in the pathogenesis of ALD. Specifically, CXCL9 and CXCL10 appear to be associated with disease progression and poor short-term outcomes, but they did not outperform established clinical scores in this study. Our results indicate that CXCLs reflect the severity of hepatic inflammation and dysfunction that increase mortality, correlating strongly with traditional scores. A future prognostic model might combine existing indices with CXCLs to see if it improves risk stratification. Further research is essential to translate these insights into clinical settings. The incorporation of CXC chemokine profiling into medical practice could aid in patient risk stratification, and CXC receptor antagonists may represent a promising area of potential therapeutic intervention because immune cell interactions emerge as critical therapeutic targets in ALD. Elucidation of the C-X-C motif chemokine significance in the context of alcohol-induced liver injury and its life-threatening complications warrants future therapy development with great potential to mitigate the adverse ethanol impact on the immune system.

## Figures and Tables

**Table 1:** Comparison of demographic data of the study and control groups.

Variable	Patients with ALD *n* = 63	Controls *n* = 25	*p*
Age median (25–75 percentiles)	49.00 (41.00–58.00)	45.00 (33.15–50.25)	0.0793
Gender *n* (%)			
Males	44 (69.84)	16 (64.00)	0.5978
Females	19 (30.16)	9 (36.00)	

**Table 2:** Comparison of characteristics of the ALD cohort based on their gender ^a^.

	ALD Study Group, n = 63		*p*
	Females with ALD *n* = 19	Males with ALD *n* = 44	
	Median 25–75 Percentiles	Median 25–75 Percentiles	
Age [years]	53.00 38.00–57.75	47.00 41.00–59.00	1.0000 ^#^
ALT IU/L	41.00 30.00–66.00	50.00 28.75–93.75	0.6801 ^#^
AST IU/L	106.00 75.50–162.00	127.50 81.00–174.00	0.5130 ^#^
ALP IU/L	136.00 99.00–217.25	130.50 95.00–227.00	0.8563 ^#^
GGT IU/L	415.00 297.00–775.00	365.00 185.00–679.25	0.3883 ^#^
T-bilirubin [mg/dL]	2.10 1.25–6.28	2.90 1.55–8.95	0.5291 ^#^
Albumin [g/dL]	2.90 2.57–3.79	2.96 2.27–3.58	0.4100 ^#^
INR	1.47 1.13–1.66	1.39 1.18–1.72	0.9821 ^#^
Na [mEq/L]	137.00 136.00–139.00	136.00 133.00–140.00	0.4198 ^#^
Creatinine [mg/dL]	0.80 0.70–1.00	0.80 0.70–1.00	0.9152 ^#^
HGB [g/dL]	12.20 11.77–13.95	11.50 10.50–12.70	0.1139 ^#^
RBC [×10^6^ cells/uL]	3.84 3.27–4.14	3.44 3.02–3.86	0.2397 ^#^
WBC [×10^3^ cells/uL]	7.87 5.57–9.63	7.02 4.75–9.75	0.6586 ^#^
NEU [×10^3^ cells/uL]	4.91 3.64–8.14	4.62 2.97–7.87	0.7081 ^#^
NLR	3.87 2.18–8.52	4.22 3.06–6.84	0.6750 ^#^
PLT [×10^3^ cells/uL]	153.00 123.00–225.25	127.00 82.50–167.50	0.1266 ^#^
CRP [mg/L]	29.61 3.58–69.23	20.98 6.24–39.82	0.5066 ^#^
CTP class	A = 5 B = 9 C = 5	A = 11 B = 19 C = 14	0.9073 *
mDF score	16.48 2.83–30.67	16.92 8.35–35.15	0.7531 ^#^
MELD-Na score	14.00 8.75–19.50	16.00 10.00–22.50	0.5485 ^#^
MELD 3.0 score	16.00 12.25–22.25	17.00 11.50–23.50	0.6801^#^

^a^ ALD-alcohol-associated liver disease, ALP—alkaline phosphatase; ALT—alanine aminotransferase; AST—aspartate aminotransferase; CRP—C-reactive protein; CTP-Child–Turcotte–Pugh; GGT—gamma-glutamyl transpeptidase; HGB—hemoglobin; INR—international normalized ratio; mDF—modified Maddrey’s discriminant function; MELD—Model of End-Stage Liver Disease; *p*—the level of significance, NEU—neutrophils; NLR—neutrophil to lymphocyte ratio; RBC—red blood cells; T—bilirubin-total bilirubin; WBC—white blood cells; ^#^ Mann–Whitney test; * Kruskal–Wallis test.

**Table 3:** Hepatic-related complications in ALD patients.

Type of Complication	ALD Patients *n* = 63	
	Complication Present *n* (%)	Complication Absent *n* (%)
Ascites	40 (63.49)	23 (36.51)
Hepatic encephalopathy	2 (3.17)	61 (96.83)
Esophageal varices	34 (53.97)	29 (46.03)
Poor 30-day outcome/death AH group (*n* = 16) no AH group (*n* = 47)	7 (11.11) 6 (37.50) 1 (2.13)	56 (88.89) 10 (62.50) 46 (97.87)

**Table 4:** Comparison of CXCL chemokine concentrations in patients with ALD and individuals from the control group *.

Chemokines pg/mL	ALD Total Group *n* = 63 (a)	ALD Survivors *n* = 56 (b)	ALD Non-Survivors *n* = 7 (c)	Controls *n* = 25 (d)	*p* a vs. d	*p* b vs. c	*p* b vs. d	*p* c vs. d
MIG_CXCL9	86.75 32.59–292.50	78.188 (56.99–113.07	255.19 (177.13–265.47)	59.14 (40.94–88.62	0.0047	0.0061	0.0130	0.0030
IP10_CXCL10	200.56 54.11–515.81	195.42 (114.10–321.90)	459.35 (243.93–492.58)	83.86 (74.79–113.09)	<0.0001	0.0108	0.0001	0.0001
CXCL16	2.93 1.76–7.50	2.78 (2.16–3.68)	3.15 (2.58–3.74)	1.67 (1.33–1.88)	0.0001	0.1649	<0.0001	<0.0001

* Median (25–75 percentiles), Mann–Whitney test.

**Table 5:** Logistic regression analysis for selection of independent predictors of poor 30-day outcomes in ALD patients ^a^.

Variable	Coefficient	Std. Error	Wald	*p*	Model’s AUC	Std. Error	95% CI
MODEL I							
MIG_CXCL9	0.0052315	0.0046553	1.2629	0.2611	0.972	0.0190	0.896–0.997
IP10_CXCL10	0.0071278	0.0048025	2.2027	0.1378			
CXCL16	−0.61311	0.55358	1.2267	0.2681			
MELD 3.0	0.40483	0.16060	6.3540	0.0117			
Constant	−13.07269	4.91852	7.0642	0.0079			
MODEL II							
MIG_CXCL9	0.0062316	0.0050746	1.5080	0.2194	0.977	0.0171	0.903–0.998
IP10_CXCL10	0.0077294	0.0049007	2.4876	0.1147			
CXCL16	−0.51124	0.52848	0.9358	0.3333			
MELD-Na	0.46669	0.20371	5.2484	0.0220			
Constant	−14.76076	6.21374	5.6430	0.0175			

^a^ AUC—Area under the curve. CI—Confidence interval, Std. Error—Standard error.

## Data Availability

The original contributions presented in this study are included in the article. Further inquiries can be directed to the corresponding authors.

## Abbreviations

The following abbreviations are used in this manuscript:

AH	Alcohol-associated hepatitis
ALD	Alcohol-associated liver disease
ALP	Alkaline phosphatase
ALT	Alanine aminotransferase
AMA	Anti-mitochondrial antibodies
AST	Aspartate aminotransferase
AUC	Area under the curve
AUDIT-C	Alcohol use disorders identification test-consumption
CI	Confidence interval
CRP	C-reactive protein
CTP	Child–Turcotte–Pugh
CXCL	C-X-C motif ligand
CXCR	CXC chemokine receptor
DAMP	Damage-associated molecular pattern
EASL	European Association for the Study of the Liver
GGT	Gamma-glutamyltransferase
HGB	Hemoglobin
INR	International normalized ratio
IP-10	Interferon gamma-induced protein-10 (CXC10)
MASLD	Metabolic dysfunction-associated liver disease
mDF	Modified Maddrey’s discriminant function
MELD	Model of end-stage liver disease
MIG	Monokine induced by gamma interferon (CXC9)
NAFLD	Non-alcoholic fatty liver disease
NEU	Neutrophils
NK	Natural killer cell
NKT	Natural killer T cell
NLR	Neutrophil to lymphocyte ratio
ROC	Receiver operating characteristic
WBC	White blood cells
